# Malware Detection in Internet of Things (IoT) Devices Using Deep Learning

**DOI:** 10.3390/s22239305

**Published:** 2022-11-29

**Authors:** Sharjeel Riaz, Shahzad Latif, Syed Muhammad Usman, Syed Sajid Ullah, Abeer D. Algarni, Amanullah Yasin, Aamir Anwar, Hela Elmannai, Saddam Hussain

**Affiliations:** 1 Department of Computer Science, Shaheed Zulfikar Ali Bhutto Institute of Science and Technology, Islamabad Campus, Islamabad 44000, Pakistan; 2 Department of Creative Technologies, Faculty of Computing and Artificial Intelligence, Air University, Islamabad 44000, Pakistan; 3 Department of Information and Communication Technology, University of Agder (UiA), N-4898 Grimstad, Norway; 4 Department of Information Technology, College of Computer and Information Sciences, Princess Nourah bint Abdulrahman University, P.O. Box 84428, Riyadh 11671, Saudi Arabia; 5 School of Computing and Engineering, The University of West London, London W5 5RF, UK; 6 School of Digital Science, Universiti Brunei Darussalam, Jalan Tungku Link, Gadong BE1410, Brunei

**Keywords:** Internet of Things, malware detection, CNN, LSTM

## Abstract

Internet of Things (IoT) devices usage is increasing exponentially with the spread of the internet. With the increasing capacity of data on IoT devices, these devices are becoming venerable to malware attacks; therefore, malware detection becomes an important issue in IoT devices. An effective, reliable, and time-efficient mechanism is required for the identification of sophisticated malware. Researchers have proposed multiple methods for malware detection in recent years, however, accurate detection remains a challenge. We propose a deep learning-based ensemble classification method for the detection of malware in IoT devices. It uses a three steps approach; in the first step, data is preprocessed using scaling, normalization, and de-noising, whereas in the second step, features are selected and one hot encoding is applied followed by the ensemble classifier based on CNN and LSTM outputs for detection of malware. We have compared results with the state-of-the-art methods and our proposed method outperforms the existing methods on standard datasets with an average accuracy of 99.5%.

## 1. Introduction

In recent years, the usage of inter-connected smart devices also knows an Internet of things (IoTs) in our real has been increased exponentially. The IoT devices can be accessed from anywhere, from home, office and vehicles to make everyday tasks simpler [1]. These smart devices are used in health, care services, smart home, vehicular networks, smart grids, smart cities, and other industries [2,3,4,5]. These smart devices have unique characteristics, such as lighter protocols, less power consumption, compact size and weight which make them more adaptable [6,7,8,9]. Expanded dispatch of smart devices in advertise alongside declined trust about detecting gadgets has made the web of things progressively versatile. Having the two upsides and downsides, the devices connected over the internet convoys the more danger of digital threats and attacks prompting failure of the administration to more terrible conveyed rejection of administration. The huge number of these devices containing variety and heterogeneity makes it inclined to digital dangers. As of talking there no confirmed security methods to guarantee the digital safeness of these devices.

IoT infrastructure comes at the cost of dreadful security threats through various attacks, due to the fact that there is no standard supporting all smart devices and built-in security mechanisms. IoT is a capitative platform for the attackers, as it has the potential to launch almost all kinds of network attacks on the connected devices, which in severe cases could lead to some serious losses. Malware is a wonderful platform for attackers since it may use conventional harmful software and syndicate attack techniques. So far, IoTs have no such protocol, which can assure the complete security of the connected devices. Eventually, there will be a critical need to improve defences against the many changing cyberthreats and assaults.

The utilization of devices expanded as the openness of the internet turn out to be simple. Innovation in technology increases quickly to make the smart world. Numerous devices obtain access to the internet. These devices are an essential part as these devices gather and store the user’s information. The accessibility and availability of the internet through these devices make vulnerable. The vulnerable IoT devices open various security concerns that are needful to secure the device. Malware is a developing danger to computer and network systems around the globe. Since the time the malware development units and changeable infection generators turned out to be effectively accessible, making and spreading jumbled malware has gotten a straightforward issue. The digital security vendors obtain a huge number of new malware tests regularly for examination. It has become a difficult assignment for the malware analysis to distinguish if a given malware test is a variation of known malware or on the other hand has a place with another breed inside and out. Since settling on an exact choice about the idea of an obscure malware test is vital for the refreshing of mark databases and spread of the update to their clients, consequently, merchants of cyber security software products need exact malware classification methods for this purpose.

Dynamic security fix establishment on smart devices are entangled. Installation of further moderation of potential updates is not an immediate and easy task. Remote program configuration reconstruction is not efficient for the Internet of Things environment. Current malware detection and anti-virus (AV) technologies are based on static detection or signatures-based detection, i.e., (Hash comparison), which are naturally explicit to the malware for which they are composed. In this way, they will not perceive and detect a new malware that is zero-day and has not yet been observed; these systems are more likely to miss such malware samples. To address this deficiency, many have applied machine learning and AI algorithms. A great enhancement in cybercrime is performed through the internet on such devices. Different kinds of attacks are performed, and their consideration is to obtain valuable data. These attacks are Man-in-the-center assault, SQL infusion, Zero-day abuse, and so forth.

With the ever-increasing cyber threats and attacker’s or adversaries’ capabilities and resources [10], traditional machine learning algorithms are incompetent to detect complex cyber breaches due to their fixed architecture [11]. The aim of this thesis is to provide security on the devices from various attack by using advance and latest techniques that are able to detect the attack with detection rate in less time [12]. In this contrast, Deep Learning (DL) reveals the true face of cyber data, either legitimate or attack, by detecting even the small variations or changes. Thus, Deep Learning can facilitate a deeper assessment of network data and quickly identify the anomalies. Hence, a DL-driven detection mechanism that can become highly scalable, adaptive, cost-effective, and particularly, without exhausting the underlying devices, which is a novel breakthrough in cybersecurity. This investigation is planned to have an extremely profound comprehension of models dependent on techniques’ cyber threats and attacks of malwares. The proposed framework, based on hybrid deep learning and comprising of a combination of deep learning methods, is executed on a Malwares dataset for the implementation of multi-class threat detection, including information gathering, the man in the middle, Daniel of service, and Bot attacks. The various type of attacks performed on the IoT infrastructure has the potential to damage the devices concerned in fetching the user’s data.

For the detection of cyber threats in IoT infrastructure, Deep Learning is considered an optimized technique when it comes to detecting the attack over a high-speed network. The architecture of Deep Learning imitates the working of the human brain and performs based on it. For acquiring the functionalities of the human brain, the powerful algorithms are designed, such as Artificial Neural Networks (ANNs), Convolutional Neural Network (CNNs) [13,14], Recurrent Neural Networks (RNNs), Long short-term memory units (LSTMs) [15,16], and so forth, to meet the necessity of an advanced level of attack detection to meet the security concerns. The high computational power ability of DL makes the algorithms most suitable for intrusion detection frameworks in IoTs, as deep learning algorithms provide a high rate of attack detection and considered a less time frame. The research question of the proposed project is: How can an IoT device be secured from evolving cyber threats and attacks using artificial intelligence efficiently with high accuracy? The research work intends to analyse and categorize the network traffic of IoT devices for the detection of sophisticated ever-growing cyber-attacks with high detection accuracy in a low detection time. Potential users would then be able to mitigate from attacks such as video injection etc. Therefore, we propose a deep learning based model for the accurate detection of malwares in IoT devices. Our contributions in the research are as follows:

1. Many machine learning techniques, including KNN, SVM, LR and Fuzzy C-Mean have been evaluated for malware classification.
2. Evolutionary computing techniques have less computational complexity and better global optimization. Hence, GA and PSO are used for better feature selection.
3. Deep learning algorithms such as CNN and LSTM are also studied. Moreover, CNN and LSTM are combined for better accuracy.
4. To test the efficiency of our proposed algorithm, the proposed algorithms are compared with other state-of-the-art algorithms.

The rest of the paper is organized as follows: Section 2 provides a critical evaluation of the existing methods, including both machine learning and deep learning based methods; the proposed model has been presented in Section 3, followed by the results obtained by experimentation setups in Section 4; and the research is concluded in Section 5.

## 2. Related Work

We have categorized malware detection existing methods for IoT devices [12,17,18,19,20,21,22,23,24,25,26,27,28,29,30,31,32,33,34,35,36,37,38] into two methods based on machine learning and deep learning. Researchers [17,18,19,20,21,22,23,24,25,26,27] have proposed a variety of machine learning intrusion detection methods. Table 1 critically evaluates the existing machine learning methods. Mendez et al. [1] provides a multiclass classification model. The detection accuracy of the ensemble on the MQTT dataset is higher than the deep learning algorithm, which is 99.37%. Tama et al. [18] proposed an edge malware detection method using a Fuzzy and Fast Fuzzy pattern tree with SVM, KNN Random Forests, and Decision Tree classifiers. The datasets utilized by the authors include Kaggle and Vx-Heaven. The detection accuracy is 99.83% and 98.01% on both datasets. A secure architecture used as a detection model based on network traffic has been proposed by Yan et al. [19]. Deep belief networks (DBNs) and support vector machines (SVM), two ensemble models, were employed and achieved the accuracy of 94.65%.

Costa et al. [20] introduced an intrusion detection system using Support Vector Domain. To determine if network traffic is an attack or not, a novelty detection model based on SSPV-SVDD as a classifier and SMO as a solution is used. Although the suggested method successfully classified 17471 unknown network actions with a detection accuracy of 99%, the confusion matrix and ROC curve analysis is not presented. Another method [25] introduced meta-heuristic method for intrusion detection. This approach utilized the *k*-Means algorithm and meta-heuristic Firefly Algorithm.

Results were compared for multiple techniques including K-Means+ Cuckoo, K-Means + Bat, K-Means, K-Means++, Canopy, and Farthest First and achieved the accuracy of 72%. Azmoodeh et al. [39] proposed an approach to detect ransomware using power consumption. *K*-Nearest Neighbors, Neural Networks, Support Vector Machine, and Random Forest have been used in this method for classification. The proposed method hasa 96.65% detection accuracy and 89.19% precision. Pajouh et al. [22] proposed two layers dimensionality reduction method and Two-tier Classification (TDTC) classification module for detection of the malicious activity, i.e., U2R User to Root and Remote to Local R2L. The detection rate of TDTC is 84.86%, which is high, as compared with other classifiers in this paper. Guo et al. [40] proposed a two-level hybrid KNN-based technique for anomaly detection. The detection accuracy of the hybrid approach KDD99 and Kyoto University Benchmark (KUBD) is 95.76%.

An approach to detect advanced malware by analyzing the features by grouping has been proposed by Kaur et al. [24]. They have used a machine learning technique with five different classifiers: RF (random forest), LMT (Logistic model tree), NBT (Naïve Bayes Tree), FT (Functional Tree), and J48. Among these classifiers, the random forest performs an outclass with an accuracy of 97.95%. Azmoodeh et al. [21] used a semi-supervised machine learning of k-Means clustering for labels. The dataset used in this paper is the ISCX dataset. The decision tree classifier is used to detect the attack. The detection accuracy of the proposed technique is 88%. Researchers [26] have also proposed a method for detecting botnet of IoT’s, which is logistic regression. Logistic regression allows for calculating the probability that the device start association is running a bot. This method is useful to detect the botnet which imitates with brute force attack using SSH protocol. The detection accuracy is 97.3%. Zak et al. [27] proposed a framework, called DFEL, to detect the intrusion in the IoT environment. The proposed system uses Principal Component Analysis (PCA) as an evaluation algorithm for their proposed framework. The detection accuracy achieved by the proposed framework while using the UNSW-NB15 dataset is 92.52%. On the other hand, the detection accuracy of 98.86% has been achieved on the NSL-KDD dataset.

Researchers [12,28,29,30,31,32,33,34,35,36,37,38,41,42,43,44,45,46] have also proposed deep learning-based methods for intrusion detection in IoT devices. Table 2 critically evaluates the existing state of the art deep learning-based Intrusion Detection Methods for Internet of Things (IoT) devices. An anomaly detection system based on fuzzy C-means clustering, interpolation technique, and RNN model was proposed by the Hafeez et al. [28]. The suggested methodology has a 98% accuracy rate and a 0.02 false alarm rate on the Kitsune dataset. The proposed scheme’s overall accuracy is excellent; however, the model is not tested against detection time. The method proposed in [29] is based on deep learning technique and provide DGA detection. DL algorithms adopted in this research project are RNN, LSTM, GRU, CNN, and CNN-LSTM. The model gains an average accuracy of 97.9%, 98.8%, 98.7%, 97.8%, and 98.5%, respectively. A federated framework, which works on the mechanism of deep learning, is presented in [30] for the multiparty computation and security of IoT devices. The accuracy achieved in this model is approximately 56%. In this approach, almost 46% percent of attacks are not properly classified, and they can penetrate to the system and create disaster in the system. Nguyen et al. [31] proposed D’IOT, a self-learning system to detect compromised IoT. The self-generated dataset is utilized to evaluate the proposed system. The proposed system achieves a detection accuracy of 95.6%. Another method proposed by HaddadPajouhet al. [32] used LSTM to hunt IoT malware-based on Opcodes. The evaluation of the proposed model is conducted using ARM-based IoT application execution Opcodes. The feature selection technique text mining is used in this method to obtain an important feature vector from Opcode. The detection accuracy achieved in this paper is 98%.

Diro et al. [12] proposed a deep learning method for cybersecurity that enables attack detection in IoT. The deep model and shallow model have been utilized for the detection of an attack. The detection accuracy of the DM model is as high as the SM model, as DM achieves 99.20% and SM attains 95.22% on the NSL-KDD datset. The recurrent neural network-based method has been proposed by Yin et al. [33] and achieved a binary and multi-classification detection accuracy of 83.28% and 97.09%, respectively. Researchers [34] have detected the botnet by extracting the statistical-based network flow features between hosts such as packet size, duration and the standard deviation of the packet. The proposed detection method achieves an accuracy of 99%. Kudugunta et al. [35] proposed a deep learning-based LSTM architecture that is used to exploit the content and metadata for detecting the bots. The accuracy achieved by this method is around 90%, which is still too low to detect the attack, especially on a network. Azmoodeh et al. [21] presented a deep learning-based Internet of Battlefield (IoBT) detection method to detect the attack by device operational code (Opcodes) sequence. The Opcodes transmute into vector space and the deep learning approach is applied to classify benign and malicious applications. The algorithm used in this paper is the Convolutional Neural Network with Adaboost classifier. The proposed detection method achieves 99.68% accuracy with 98.59% precision, 98.37% recall, and 98.48% f1-score.

McDermott et al. [36] proposed the novel approach of packet-level detection in IoTs and network by implementing deep learning using the Bidirectional LSTM to evaluate. In this paper, Mirai botnet traffic and normal IoT traffic are used and are generated in this paper through the experiment. The resultant detection accuracy of each class, i.e., Mirai, UDP, and DNS are 99%, 98%, and 98%. The graph of training loss is shown. Without knowing the trace generation and bandwidth of the mobile, Xiao et al. [37] presents malware detection technique for mobile devices using Q learning for the best offloading. The malware detection game in the time-variant wireless network is examined, and the author explores the Nash equilibrium (NE) of the static malware detection game. Zhao et al. [38] proposed an intrusion detection method by utilizing a deep learning algorithm deep belief network (DBN) and probabilistic neural network (PNN). The dataset considered in this paper is KDDCUP99, which evaluates the performance of the proposed method and achieved an accuracy of 99.14%.

## 3. Proposed Method

Deep learning is one of the innovations in cybersecurity. Besides that, it has the potential to solve security issues [10]. The concept of the Internet of Things arises as access to the internet turns out to be simple. Innovation in technology and energy efficiency are leading the world to the smart and intelligent embedded environment [47]. IoT, the lashing strength behind home computerization, smart cities, modern health systems, and advanced manufacturing are also compelling targets for the prevalent sophisticated cyber threats. To quickly and effectively identify increasingly complex malware, the study aims to design a platform that is scalable, economical, and effective for the ecosystem’s underpinning smart and automated systems.

Due to low computational power ability and memory constraint in smart devices, unlike smartphone and computer systems, the smart devices are not self-sufficient to execute the intrusion detection solutions on every individual device. They also lack in proper infrastructure to perform different advance techniques to not risk the devices from sophisticated malware threats and the memory constraint for storing the ever-increasing instances of malware signatures. Light weight malware detection framework helps to insert any security update and even gather the performance information of the device if any action is required to take and product to execute for improvement of performance. Due to the architectural difference, the capabilities of the smart devices on the internet highlights the need of a multi-layered distributive approach for malware detection. As the smart devices have limitations of less capabilities to handle information, the network is very open to security, information leakage and privacy violation. The multi-layered architecture approach helps to deal with the data generation and communication in the smart devices, making it robust. Additionally, in the network, the data is generated through billions of devices, processed and saved through different techniques and even also transmitted through different and diverse locations. However, a single layer architecture is not capable enough to demonstrate optimized performance due to the restriction of the scope of components. As the multi-layered structure is distributed throughout and across the system, it allows different tasks and processes to execute and run at different levels of hierarchy, for example from composite to simple problems that are solely dependent on the current scenario.

In the smart world, the longevity levels depend on the consumer’s devices. Such as in smart cities, the time span of a device is approximately around 10 to 20 years. Thus, for malware detection solution, the life cycle of these should have the capability to learn the better concept of the identification of cyber threats and attacks from previous logics, instances, and logics without any human interference. The total life circle of smart devices, including smart TVs, microwave ovens, washing machines and even refrigerators, usually work for very long time periods and perform their predefined tasks without the manufacturer assistance. There comes the longevity, which is quite important to collect the demand and requirements for the smart devices in the automated environment for improvement in the management of them. It is a difficult task for agencies to monitor the devices deployed in the environment for a long time.

**Figure 1:**
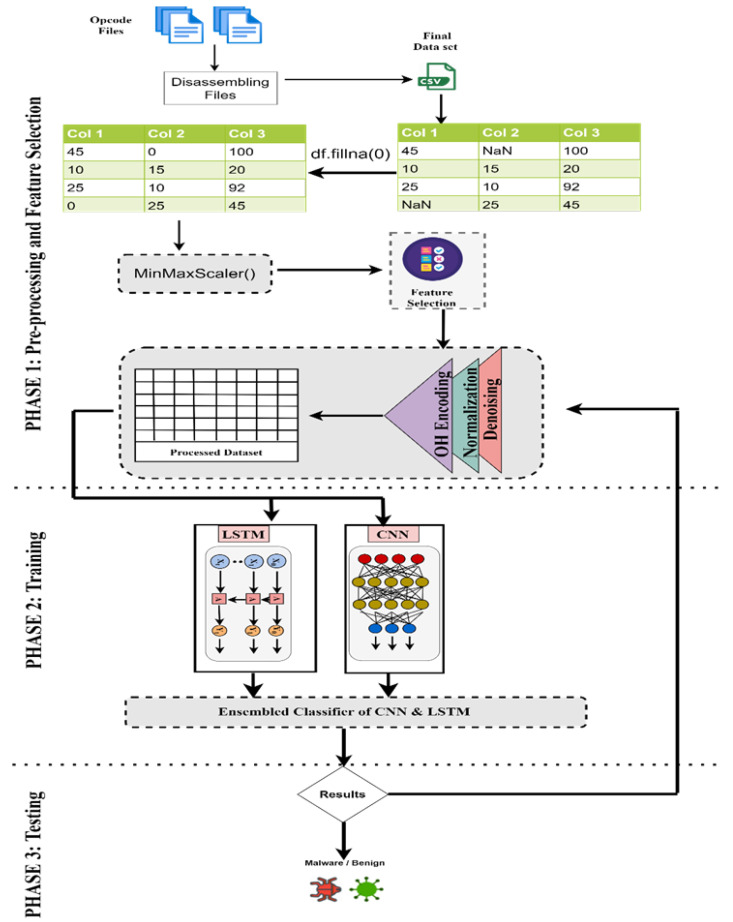
Proposed system model for hybrid deep learning malware detection algorithms.

**Figure 2:**
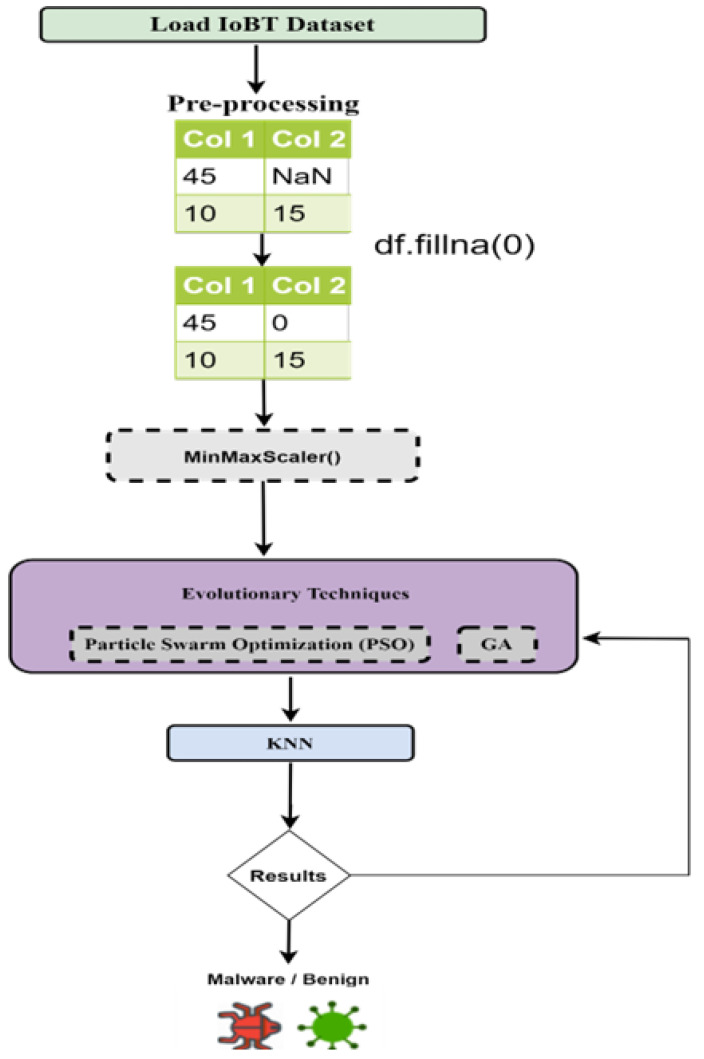
Proposed flow for hybrid machine learning malware detection algorithms.

For the detection of malwares in the data, Figure 1 shows the proposed framework for the implementation of the detection of malwares using LSTM and CNN. The model is simulated on the IoBT malwares Dataset. Opcode files were disassembled for creating the dataset. The dataset file is used as the input for the model. The raw data are passed through the processing and feature selection phase and then passed to the classifier to detect benign and malicious classes of malware. Figure 2 shows the proposed model for the implementation of Machine Learning algorithms along with evolutionary computing techniques. The input data file was passed on to the classifier after the pre-processing and feature selection phase. Based on the analysis of the resulting data, the deep learning enabled the Intrusion Detection-based framework, which can facilitate smart devices for automated classification of network traffic in the real-world and detect malicious flow, not passing it for further processing.

### 3.1. Dataset

**Figure 3:**
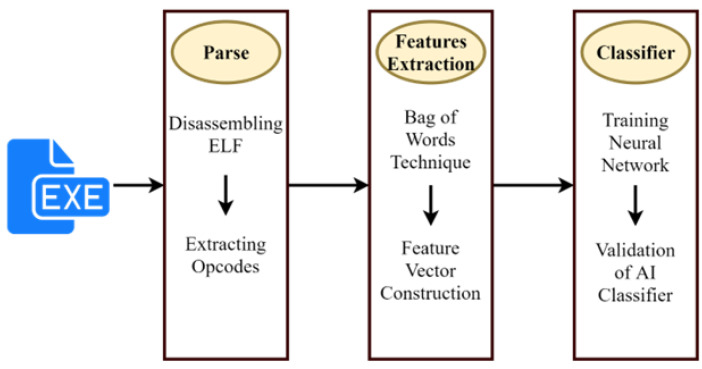
Opcode extraction.

For security reasons, the malware in the dataset is already disassembled and their bytecode is provided. To extract the byte code of all benign files in the dataset, we have used radare to disassemble all benign files. Furthermore, after disassembling all the executables and getting bytecode for good wares and malware, a bag of word technique is used to create a dataset out of bytecodes. For the selection of features, some critical assembly language instructions have been taken as features. The Figure 3 shows the extraction of the opcode.

For a full review, we used a publicly available dataset of IoBT malwares to present state-of-the-art malware [11]. The dataset includes both benign and malware samples (i.e., 128 malwares and 1089 benign files) in raw format (i.e., benign samples are ELF (Executable Likable Format) files, whilst malware samples are delivered in the form of opcode sequences.). The ELF samples are then decompiled when all of the files have been extracted. The files are disassembled using a Python script to generate opcode sequences using Radare2. Radare2 is a powerful binary analysis and reverse engineering framework. The files have been deconstructed and are now available in raw format. We use the bag of words methodology to create a feature vector. Finally, the feature vector is used to classify malware and benign samples (Table 3). On crucial opcode instructions chosen from all generated opcode sequences, the bag of words approach is used. Finally, the selected opcodes are used to generate a 284-word dictionary. Imports, instructions, libraries, and string are the types of vectors that arise.

In the pre-processing phase, the dataset is taken as a CSV file an input parameter that reconstructs data instances into deep learning classifiers compatible with training and testing. The pre-processing of the dataset includes the import method, handling missing and infinity values, normalization of the dataset, and reshapes method. The pre-processing of the dataset is required when you have to refine the dataset to detect the cyber-attacks. Removing the unnecessary data from the dataset helps to improve the attack detection rate in smart devices that are merged on the network day by day.

### 3.2. Feature Selection

As smart devices contained numerous features, the preprocessing of information is required to recognize significant and most applicable features. The point of features choice procedures to apply before classification is to improve malware identification precision and diminish the computational unpredictability for preparing a profound learning model. The observations of recent works are in favor of combining the output of different feature filtration methods to enhance the performance of the classifier, as sometimes the individual identity of feature is weak but strong when it comes to a group. In our work, we have utilized the features which are top ranked by the feature selection technique Particle Swarm Optimization (PSO). To achieve efficient and accurate results, we have performed feature filtration techniques to obtain significant features. As shown in Table 4 after performing experiments, we have chosen the top 64 features: The 64th feature is a class label.

### 3.3. Classification

The classifier class of our proposed deep learning architecture is defined. To make the malware detection framework more robust, the dropout layer has been utilized. The main use of the dropout layer is to prevent the model from overfitting. The steps and their respective actions are defined as follows:

1. Import the Keras library;
2. Initialize of neural network;
3. Add the input layer with defined dimensions and dropout layer;
4. Add hidden layers with the number of neurons and set the dropout layer value;
5. Add the output layer with the total number of label classes as neurons.

### 3.4. Deep Neural Networks

Deep neural networks (DNNs), additionally called Deep feed-forward systems or feedforward neural systems or multi-layer perceptron’s (MLPs), are considered as an ideal arrangement for supervised learning. DNNs are one kind of Deep learning design, notwithstanding repetitive recurrent neural systems (RNNs), and furthermore, convolutional deep neural systems (CNNs). This exploration centers on the utilization of DNNs for the errand of system interruption identification. DNNs can speak to elements of expanding unpredictability, by considering more layers and more units per layer in a neural system. With regards to NIDSs, DNNs can be utilized to find examples of favorable and pernicious traffic covered up inside huge measures of organized log information. A deep, fully linked neural network is observed in the diagram, as every one of the neurons in the input layers are associated with each neuron at each progressive layer. The last is an increasingly disentangled portrayal of a two-layer completely associated neural system. These figures pass on normal documentation utilized for representing deep neural systems and will be the documentation followed for the remainder of this work. Nodes represent inputs, and edges speak to weight or biases.

### 3.5. Long Short-Term Memory (LSTM)

Long Short-Term Memory (LSTM) is an expansion of Recurrent Neural Network. LSTM considered the idea of utilizing the gates for units. One significant problem with RNN is it cannot learn the setting data over a drawn-out range of time because of the disappearing slope issue, which is, during a long worldly hole (for example time from when info is acquired to when the information is utilized to make a forecast). Subsequently, RNNs are unequipped for gaining from long-separation conditions [48]. One response to the issue of the evaporating angle is an LSTM plan. It turns away the issue of the disappearing slope, and in this manner, allows the maintenance of the prolonged time of setting. The cell has input in LSTM $x_{t}$ and the hidden $h_{t}$. During the preparation stage, the LSTM cell additionally considers the input state, Ct, the cell hidden state, $h_{t}$ and the past cell state, $C_{t-1}$. The gate mechanism permits LSTM to manage previously mentioned long-separation conditions. The working of each gate that is signified as $i_{t}$, denotes the vector for input or updated gate, $o_{t}$ is the activation vector for the output gate and $f_{t}$ is the activation vector for the forget gate. All the three gates are meant by shaded confines. σ is the activation function, *W* is the weight of the neuron, $X_{t}$ is the input, $h_{t-1}$ is referred to as the input from the previous cell, ht is the final output of the cell, and lastly, *b* is the bias added on each neuron. In our case of malware classification, all of the features were passed in an input vector for each instance, i.e., *x* = $x_{1}$ + $x_{2}$ + … $x_{t}$.

## 4. Results and Discussion

A comparison of the proposed schemes is performed with the existing malware detection frameworks. The results demonstrated that GA-KNN and PSO-KNN performed better as compared with other machine learning algorithms. Table 5, Table 6, Table 7 and Table 8 shows the results achieved with different experimental settings (Appendix A). The PSO-KNN, GA-KNN and SVM has achieved an accuracy of 99.97%, 99.4% and 98.08%, respectively, which is significantly better in comparison with [32]. The logistic regression accuracy is 93.16%. The accuracy of fuzzy c-mean is the lowest in comparison with the other ML algorithms, and is 87.92%.

The proposed GA-KNN and PSO-KNN perform better due to the non-parametric nature of KNN and better optimization capabilities of GA and PSO. The main aim for the application of the algorithms was to achieve better accuracy for the detection of malwares threats; to achieve this purpose, different machine learning algorithms are used. Among them, KNN perform better. The accuracy is further enhanced by using GA and PSO for feature selection. The Table 5 demonstrated 10-fold cross validation accuracy for ML algorithms. GA-KNN generally performs better than the others in most folds. However, in some cases, Logistic regression performs better. The PSO-KNN, logistic regression, SVM and GA-KNN has achieved a precision of 99.89%, 99.8%, 97.5% and 97.3% respectively. The Table 6 represents a 10-fold cross validation precision for ML algorithms. Logistic Regression has a minimum of 90% precision in the third fold and maximum of 99.8% with the first, second, fourth and fifth fold. SVM has the minimum precision. The PSO-KNN, logistic regression, SVM and GA-KNN has achieved precision 99.89%, 99.8%, 97.5% and 97.3%, respectively. The Table 7 represents a 10-fold cross validation recall for ML algorithms. The PSO-KNN, GA-KNN, SVM and logistic regression has achieved a recall of 99.87%, 98.13%, 86.66% and 44.44%, respectively.

The PSO-KNN, logistic regression, SVM and GA-KNN has achieved a recall of 99.87%, 98.13%, 86.6% and 44.4%, respectively. The Table 8 indicates 10-fold cross validation F1-Score for ML algorithms. The PSO-KNN, GA-KNN, SVM and logistic regression has achieved F1-Score 99.83%, 96.24%, 91.76% and 61.53%, respectively. In Table 9, the hybrid LSTM and Hybrid CNN has achieved the accuracy of 99.5%, 99.1%, respectively. Moreover, deep learning algorithms are used because the dataset used in this study is unstructured. To remove the biasness in the dataset, we have performed a 10-fold cross validation technique. The 10-fold cross validation technique is basically a technique in which the dataset is divided into 10 different sets and the training is performed on 9 sets and tested on the 10th set. This process executes again and again until 10 sets are not completed. The Table 10 represents the 10-fold cross validation accuracy, precision, recall and F1-Score of DL algorithms. The Hybrid CNN, Hybrid CNN-LSTM and hybrid LSTM has achieved a precision of 99.9%, 99.8% and 99.7%. The Hybrid CNN, Hybrid CNN-LSTM and hybrid LSTM has achieved a recall of 100%, 100% and 99.7%. The Hybrid CNN, Hybrid CNN-LSTM and hybrid LSTM has achieved F1 - Score of 100%, 100% and 99.7%.

**Figure 4:**
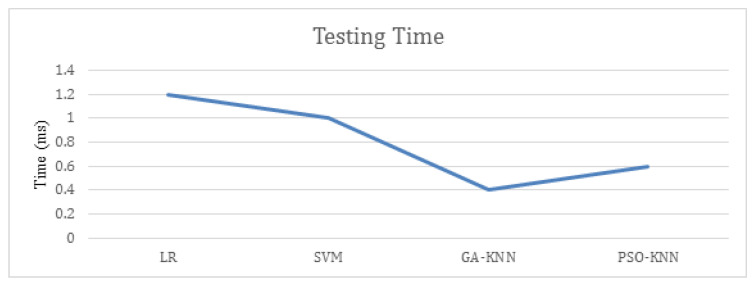
Testing time of machine learning algorithms.

**Figure 5:**
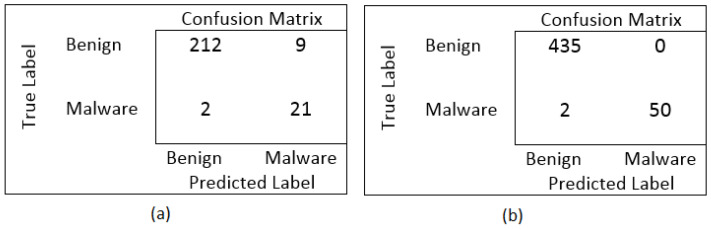
(**a**) Confusion matrix for Hybrid CNN-LSTM (**b**) Confusion matrix for PSO-KNN.

The testing time for our proposed and other contemporary algorithms are also calculated and presented in Figure 4. Deep learning classifiers are very optimal to identify the ever evolving sophisticated cyber threats but if the scheme will take a lot of time in identification so the enterprises cannot rely on it. Thus, the time complexity is calculated and compared with other experimented classifiers. For the enhanced evaluation of the proposed machine learning and deep learning algorithm to identify false occurrences that include true positives and negatives along with false positives and negatives. The confusion matrix of hybrid deep learning model and PSO-KNN is shown in Figure 5. It could be observed that the cumulative false positive and negative for deep learning hybrid model are 11 samples, whereas for PSO-KNN it is only two samples.

## 5. Conclusions and Future Work

The exponential increase in smart devices has brought great smart automation and exceptional revenue creation. Because of the digital landscape, and the integration of heterogeneous ecosystem in human lives are making this infrastructure complex and also challenging to protect it. Moreover, due to providing feasibility and easy access to digital assets, these devices are prone to lethal cyber-attacks, threats, and vulnerabilities. For securing these devices from attacks, it is a very prime need to develop, design, and implement some cyber threat and attack mechanisms, which can assure that the smart devices are not compromised from this type of threats and attacks. In this regard, different threat identification schemes have been presented in the past, which includes firewalls, antiviruses and machine learning and deep learning-based intrusion detection mechanisms. However, the static detection mechanisms are not very capable to identify attacks, as these techniques are rule-based and the new and prevalent attacks are methods to bypass network and device security.

The designed algorithms for machine learning techniques are also static and provide good identification accuracy, but when it comes to huge data, their performance accuracy lacks. In this work, this is the identification of IoBT malwares. Several machine learning and deep learning classifiers are implemented. For providing security from lethal and sophisticated malwares, we have proposed a hybrid of a deep learning technique, named the Convolutional Neural Network-Convolutional Neural Network (CNN-CNN). The proposed technique achieves a very promising detection accuracy of around 99%. For the comprehensive evaluation, we have also experimented other hybrid classifiers and other machine learning algorithms. The time complexity of the presented CNN-CNN is also very good, as compared to other schemes. Limitation of this research include a consideration of only static malware detection, using multiple instances of a single dataset, nonconsideration of CPU and RAM, and the proposed method has not been tested in the real time environment.

In future, we have planned to extend the work with the help of multiple experiments by combining the both machine learning and deep learning approaches for a comprehensive malware detection method. Currently, more than a thousand instances of the single dataset have been used for training and testing, and in future, multiple datasets can be used for experimentation and increasing the robustness of the proposed method on the test dataset. Static malware detection method has been used in this research, whereas, dynamic malware detection can be conducted in the future. Moreover, through creating a simulated ecosystem, the dataset can be created and then experimented using different AI-enabled techniques. Real time malware detection can also be tested in future on the IoT devices with live streams of data with the help of the proposed method. RAM and CPU also plays a vital role in the IoT devices; therefore, we would also consider these facts in the experimentation for malware detection in IoT devices in the future along with the testing time. Ablation techniques for consideration of the features and selection of a classifier can also be performed to increase the robustness of the method.

## Figures and Tables

**Table 1:** Critical evaluation of existing state-of-the-art machine learning-based intrusion detection methods for Internet of Things (IoT) devices.

Method	Dataset	Algorithm	Results	Limitations
[17]	MQTT Dataset	Deep learning and machine learning-based Multiclass classification model that is used to feed into intrusion detection system using RF, Boosting Gradient, SVM, and LSTM	Accuracy: 99.37 Precision: 96 Recall: 95.67 F1-score: 95.67	The testing time is not mentioned. Confusion matrix and ROC-Curves are missing
[18]	Kaggle and Vx Heaven datasets.	Machine learning-based Edge malware detection is considered an investigation into its potentiality for the proposed solution Fuzzy and Fast Fuzzy pattern tree	Accuracy: 98.01 Precision: 99 Recall: 99.10 F1-score: 99.10	The value of FPR, FDR, FNR, FOR, and time complexity is not mentioned
[19]	SCADA network traffic	Machine and deep learning-based secure architecture ICS network that is used as a detection model based on two ensemble model deep belief network (DBN) and SVM	Accuracy: 94.65	Need to consider feature selection techniques to help to gain detection accuracy. Lack in calculating various parameters such as precision, recall FDR, F-1 score
[20]	NSL, KDD	Machine learning-based intrusion detection system (IDS) based on SSPV SVDD as classifier and SMO	Accuracy: 99 Precision: 93 Recall: 92.62 F1-score: 92.68	Paper lack in the experimental part. The paper only considered the detection accuracy.
[21]	NSL, KDD	Machine learning-based novel metaheuristic approach to build an intrusion detection system	Accuracy:72 Precision:69 Recall:68 F1-score:68	The detection accuracy is too low. Need to use some feature selection technique
[22]	Self Generated	Machine learning-based method to detect the ransomware using power consumption with KNN, MLP, DT	Accuracy: 95.65 Precision: 89.19 Recall: 85 F1-score: 85	The dataset is not well enough to detect an attack. Need to a utilized deep learning algorithm.
[23]	NSL, KDD	Machine learning-based novel model for intrusion detection based Two-layer Dimension Reduction and Two-tier Classification (TDTC) classification module using KNN	Accuracy: 84.86 Precision: 80.37 Recall: 79 F1-score: 79	The detection accuracy is very low overall. Need to use a deep learning technique.
[24]	Malicia	Machine learning-based malware detection system	Accuracy: 97.95	Detect the advanced malware by analyzing the features by grouping them.
[25]	ISCX	Machine learning-based K-mean Clustering Decision Tree	Accuracy: 88 Precision: 85 Recall: 83 F1-score: 83	Classify the traffic by using a distributed framework.
[26]	Self Generated	Machine learning-based Logistic Regression	Accuracy: 97.3 Precision: 95.49 Recall: 93.10 F1-score: 93.10	Detect the botnet on IoT’s initiate with brute force attack using SSH protocol.
[27]	NSL, KDD	Machine learning based on a framework DFEL, to detect the intrusion in the IoT environment.	Accuracy: 98.86 Precision: 96 Recall: 95.32 F1-score: 93	The detection accuracy is low. Need to consider feature selection. Techniques help to gain detection accuracy. Confusion matrix and ROC-Curve are missing

**Table 2:** Critical evaluation of existing state-of-the-art deep learning-based Intrusion detection methods for Internet of Things (IoT) devices.

Method	Dataset	Algorithm	Results	Limitations
[28]	Kitsune	Proposed an anomaly detection system based on recurrent neural network model	Accuracy: 98%	Testing time is not calculated.
[29]	DS1-D1, DS1-P	Presented an IoT attack detection scheme	Accuracy: 98.8%	Old dataset used.
[30]	Self-generated	DL-based federated technique is presented for multiparty computation with the security of IoT devices	Accuracy: 56%	Algorithm is unable to achieve promising detection accuracy.
[31]	Self-generated	Proposed Self-Learning system D’IOT, an autonomous self-learning system to detect compromised IoT devices	Accuracy: 95.6% Precision: 92.10% Recall: 89% F1-score: 89	The detection accuracy is low. Need to consider feature selection techniques to help to gain detection accuracy.
[32]	ARM-based IoT application execution Opcodes	Proposed Deep learning-based Long-short-Term-Memory (LSTM) algorithm to hunt IoT malware based on Opcodes	Accuracy: 98%	A very small dataset has been used.
[12]	NSL-KDD	Deep learning-based cybersecurity that enables the attack detection in IoT using deep model (DM) and shallow model (SM)	Accuracy: 99.20% Precision: 95.22% Recall: 93% F1-score: 93%	The used dataset belongs to the traditional network landscape and has no IoT traffic and attacks
[33]	NSL-KDD	Deep learning-based intrusion detection using recurrent neural networks (RNN-IDS).	Accuracy: 97.09% Precision: 83.28% Recall: 81% F1-score: 81%	Testing time required for detection is high.
[34]	CTU-13	Deep learning-based method to detect the botnet by extracting the statistical-based network flow features between hosts such as packet size, duration, and the standard deviation of the packet using DNN	Accuracy: 99%	Testing time is not calculated.
[35]	Obtain dataset from Cresci and collaborators	Deep learning-based model to exploit the content and metadata for detecting the bots using LSTMs	Accuracy:90%	The detection accuracy is low. Need to consider feature selection techniques for better accuracy. Testing time is not calculated. The confusion matrix and ROC-Curves and values of FPR, FDR, FOR, FNR are missing
[36]	Mirai botnet data and Self generated data	Proposed Deep learning-based Novel approach of packet-level detection in IoTs and network by implementing Bidirectional LSTMs	Accuracy: 99 Precision: 98% Recall: 95.45% F1-score: 95.45%	Testing time is not calculated. The confusion matrix and ROC-Curve analysis is missing
[37]	Real time environment	Malware detection scheme for mobile devices with Q learning for optimal offloading	Accuracy: 67%	Accuracy is too low.
[38]	DARPA KDDCUP99	Deep learning-based intrusion detection method by utilizing deep belief network and PNN.	Accuracy: 99.14%	The value of FPR, FNR, FDR and FOR are missing

**Table 3:** Features of the generated dataset for experimentation.

Sr. No.	Feature Name	Type	Sr. No.	Feature Name	Type
1	call	Instructions	12	str.FlushProcessWriteBuffers	Strings
2	push	Instructions	13	str.ComparreStringEx	Strings
3	pop	Instructions	14	str.EnumSystemLocalesEx	Strings
4	leave	Instructions	15	str.IsValidLocaleName	Strings
5	shl	Instructions	16	str.Kernel32.dll	Strings
6	cmp	Instructions	17	sym.imp.KERNEL32.dll HeapAlloc	Imports
7	eax	Instructions	18	sym.imp.str_str	Imports
8	jbe	Instructions	19	sym.imp.strlen	Imports
9	str.GetThreadLocale	Strings	20	sym.imp.memcpy	Imports
10	str.RuntimeErrorProgram	Strings	21	sym.imp.free	Imports
11	str.FlsSetValue	Strings	22	sym.imp._fprintf_chk	Imports
			23	sym.imp.KERNEL32.dll_GMH	Imports

**Table 4:** List of selected features using multiple feature selection techniques.

Algorithm	Selected Features Count	Feature Index
PSO + CfsSubsetEval	64	2, 14, 15, 18, 19, 20, 28, 39, 41, 44, 45, 47, 63, 72, 90, 92, 94, 102, 116, 117, 128, 130, 140, 146, 150, 152, 156, 160, 161, 165, 169, 175, 180, 182, 206, 207, 209, 210, 211, 212, 213, 215, 218, 219, 225, 226, 232, 233, 236, 237, 240, 244, 249, 253, 255, 256, 258, 262, 265, 269, 270, 275, 277, 283
PSO + Classifiersubseteval	9	9, 55, 61, 88, 169, 198, 222, 230, 244
PSO + WrapperSubsetEval	10	9, 34, 44, 49, 54, 113, 146, 234, 237, 238
PCA + Ranker	29	1, 2, 3, 4, 5, 6, 7, 8, 9, 10, 11, 12, 13, 14, 15, 16, 17, 18, 19, 20, 21, 22, 23, 24, 25, 26, 27, 28, 29

**Table 5:** 10-Folds’ accuracy comparison for machine learning algorithms.

Result of *k*-Fold	Logistic Regression	SVM	GA-KNN	PSO- KNN
Average	99.36	99.08	98.49	98.44
Variance	0.6864	0.5376	1.8809	4.8464

**Table 6:** 10-Folds’ precision comparison for machine learning algorithms.

Result of *k*-Fold	Logistic Regression	SVM	GA-KNN	PSO-KNN
Average	98.28	96.48	99.49	95.18
Variance	9.77	21.37	0.092	47.46

**Table 7:** 10-Folds’ recall comparison for machine learning algorithms.

Result of *k*-Fold	Logistic Regression	SVM	GA-KNN	PSO-KNN
Average	99.42	99.32	99.16	98.36
Variance	0.324	0.32	0.14	3.17

**Table 8:** 10-Folds F1-Score comparison for machine learning algorithms.

Result of *k*-Fold	Logistic Regression	SVM	GA-KNN	PSO-KNN
Average	99.38	98.36	98.31	96.3
Variance	0.31	3.59	1.41	21.91

**Table 9:** Deep learning algorithms’ accuracy comparison.

	Hybrid CNN-CNN	Hybrid CNN-LSTM	Hybrid LSTM-LSTM	[15] LSTM	[20] LSTM-RNN
Accuracy	99.1%	99.8%	99.5%	98.18%	98.33

**Table 10:** Comparison for deep learning algorithms.

Result of *k*-Fold	Accuracy		Precision		Recall		F1-Score	
	Hybrid CNN	LSTM- CNN	Hybrid CNN	LSTM- CNN	Hybrid CNN	LSTM- CNN	Hybrid CNN	LSTM- CNN
Average	99.83	99.79	99.80	99.78	99.79	99.93	99.71	99.85
Variance	0.09	0.26	0.11	0.33	0.07	0.02	0.09	0.04

## Appendix A

**Table A1:** 10-Folds’ accuracy comparison for machine learning algorithms.

Fold	Logistic Regression	SVM	GA-KNN	PSO- KNN
1	99.4	99.4	99.6	92
2	99.4	99.4	99.8	99
3	97	97	99.4	98
4	99.8	99	98.7	99.8
5	99.8	99.8	95.4	99.8
6	99.8	99.4	96.5	99.4
7	99.8	99	98.9	99.4
8	99.8	99.4	98.1	99
9	99.8	99.4	99.3	99
10	99	99	99.2	99

**Table A2:** 10-Folds’ precision comparison for machine learning algorithms.

Fold	Logistic Regression	SVM	GA-KNN	PSO-KNN
1	99.8	99.8	99.4	79
2	99.8	99.8	99	90
3	90	90	99.7	90
4	99.8	96	99.4	99.8
5	99.8	99.8	99	99.8
6	99.4	99.8	99.4	99.8
7	99.4	90	99.8	99.4
8	99.4	99.8	99.7	99
9	99.4	99.8	99.8	99
10	96	90	99.7	96

**Table A3:** 10-Folds’ recall comparison for machine learning algorithms.

Fold	Logistic Regression	SVM	GA-KNN	PSO-KNN
1	99.8	99.8	98.6	95
2	99.8	99.8	98.7	99
3	98	98	99.1	99
4	99.8	99	99.4	99.4
5	99.8	99.8	99.2	99.4
6	99.8	99.8	99	99.4
7	99.4	99.2	99.7	99.4
8	99.4	99.4	99.1	99
9	99.4	99.4	99.8	95
10	99	99	99	99

**Table A4:** 10-Folds’ F1-Score comparison for machine learning algorithms.

Fold	Logistic Regression	SVM	GA-KNN	PSO-KNN
1	99.8	99.8	99.8	84
2	99.4	99.4	99.4	95
3	98	94	98.5	95
4	99.4	97	97.8	99.4
5	99.4	99.4	99.1	99.4
6	99.4	99.4	95.6	99.4
7	99.8	98	97.6	99.8
8	99.8	99.8	98.1	97
9	99.8	99.8	99	97
10	99	97	98.2	97

**Table A5:** Comparison for Deep Learning Algorithms.

Folds	Accuracy		Precision		Recall		F1-Score	
	Hybrid CNN	LSTM- CNN	Hybrid CNN	LSTM- CNN	Hybrid CNN	LSTM- CNN	Hybrid CNN	LSTM- CNN
1	99.99	99.99	99.99	99.99	99.4	99.99	99.99	99.99
2	99.99	99.99	99.4	99.99	99.99	99.99	99.4	99.99
3	99.4	99.8	99.99	99.99	99.6	99.99	99.4	99.4
4	99.99	99.99	99.6	99.8	99.99	99.99	99.99	99.8
5	99.99	99.99	99.99	99.99	99.99	99.8	99.6	99.99
6	99.8	98.36	99.99	98.16	99.4	99.99	99.99	99.99
7	99.99	99.99	99.99	99.99	99.99	99.99	99.99	99.8
8	99.17	99.6	99.08	99.99	99.6	99.99	99.4	99.99
9	99.99	99.99	99.99	99.99	99.99	99.6	99.99	99.6
10	99.99	99.99	99.99	99.99	99.99	99.99	99.4	99.99
Average	99.83	99.79	99.80	99.78	99.79	99.93	99.71	99.85
Variance	0.09	0.26	0.11	0.33	0.07	0.02	0.09	0.04
